# Language Production and Prediction in a Parallel Activation Model

**DOI:** 10.1111/tops.12775

**Published:** 2024-11-22

**Authors:** Martin J. Pickering, Kristof Strijkers

**Affiliations:** ^1^ Department of Psychology University of Edinburgh; ^2^ Laboratoire de Parole et Langage (LPL) Aix‐Marseille Université & CNRS

**Keywords:** Language production, Prediction, Parallel architecture

## Abstract

Standard models of lexical production assume that speakers access representations of meaning, grammar, and different aspects of sound in a roughly sequential manner (whether or not they admit cascading or interactivity). In contrast, we review evidence for a parallel activation model in which these representations are accessed in parallel. According to this account, word learning involves the binding of the meaning, grammar, and sound of a word into a single representation. This representation is then activated as a whole during production, and so all linguistic components are available simultaneously. We then note that language comprehension involves extensive use of prediction and argue that comprehenders use production mechanisms to determine (roughly) what they would say next if they were speaking. So far, theories of prediction‐by‐production have assumed sequential lexical production. We therefore reinterpret such evidence in terms of parallel lexical production.

## 1 Introduction

Standard accounts of lexical access assume that speakers retrieve linguistic representations by first computing a word's meaning, then its grammar, and finally its sound (Levelt, Roelofs, & Meyer, 1999). And while it has been fiercely debated whether these linguistic components are accessed in a discrete and serial manner (Levelt et al., 1999) or in an interactive and cascaded manner (e.g., Caramazza, 1997; Dell, 1986), these accounts agree that production is broadly sequential, with each step taking tens or hundreds of milliseconds (Indefrey, 2011; Indefrey & Levelt, 2004; Sahin, Pinker, Cash, Schomer, & Halgren, 2009). But some recent accounts challenge this assumption and instead propose that word components are activated in parallel (e.g., Kerr, Ivanova, & Strijkers, 2023; Strijkers & Costa, 2016). In this article, we first present the evidence for parallel lexical access and relate it to the parallel activation model. We then turn to language comprehension and consider the implications of parallel lexical access for accounts in which comprehenders use language production to make predictions (Pickering & Gambi, 2018).

### 1.1 Lexical access in a parallel activation model

A parallel activation model of lexical access in language production, like the equivalent model in comprehension (e.g., Pulvermuller, 1999; 2018), assumes that the different linguistic components of a word are retrieved simultaneously (e.g., Kerr et al., 2023; Strijkers, 2016; Strijkers & Costa, 2016). This activation dynamic is based on an influential neurophysiological principle of brain organization, namely, Hebbian‐based learning and assembly coding. This principle assumes that neural representations that are active at the same time will bind together into a single functional unit (e.g., Buzsaki, 2010; Hebb, 1949; Singer, 2013). If for a mental event X, there is a consistent temporal correlation between the activation of neural populations A and B, then A and B will bind together in a novel, overarching assembly C that is associated with mental event X as a whole. As the meaning, grammar, and sounds of a word consistently co‐occur,^1^ through Hebbian‐based learning, they form an integrated word assembly (e.g., Pulvermüller, 1999; 2018; Pulvermüller & Fadiga, 2010). For instance, when I start uttering *“ball*,” I might initially access its meaning (*bouncing, round, toy*), then its grammar (*noun*), and then its sound (/*b/‐/ɔ/‐/l/*). But as I consistently activate *“/bɔl/*,” after activating a *“noun”* that reflects a *“bouncing round toy*,” I come to activate them at the same time as each other. So, when I come up with the intention of describing a *“ball”* and access its meaning, there is no longer a delay before activation of *“/bɔl/”—*they happen at the same time as each other because they come to form an integrated word assembly. Note that the starting point of learning is not so different from traditional models that have specific strata dedicated to meaning‐, grammar‐, and sound‐related processing. The difference emerges during development when features are bound across these meaning‐, grammar‐, and sound‐related strata to form a coherent word representation.

This account fares well in explaining recent studies showing the same, early time‐course of activation of semantic, lexical, and phonological manipulations (e.g., Fairs, Michelas, Dufour, & Strijkers, 2021; Feng, Damian, & Qu, 2021; Miozzo, Pulvermüller, & Hauk, 2015; Riès et al., 2017; Strijkers, Costa, & Thierry, 2010; 2017). For instance, in a magnetoencephalography (MEG) study of overt object naming, Strijkers, Costa, and Pulvermüller (2017) manipulated the frequency of to‐be‐uttered words and the place of articulation of the initial phoneme (e.g., coronal in “*D*onkey” versus labial in “*M*onkey”). If lexical access in language production is sequential, effects related to the lexical frequency manipulation should occur before effects related to the phoneme manipulation (since it is well‐established that lexical frequency affects lexico‐semantic processing: e.g., Caramazza, Costa, Miozzo, & Bi, 2001; Corps & Meyer, 2023; Kittredge, Dell, Verkuilen, & Schwartz, 2008; Strijkers et al., 2010). If, however, the lexico‐semantics and phonology of a word are accessed simultaneously, then the effects of the two manipulations should emerge concurrently. In accord with the latter claim, Strijkers et al. (2017) found that lexical frequency activated left inferior frontal gyrus and the mid‐temporal gyrus between 160 and 240 ms after stimulus onset, and the coronal versus labial phonemes activated the tongue and lip motor cortex in exactly the same time window.

Recently, Fairs et al. (2021) found a similar result with electroencephalography (EEG). Participants passively listened to spoken words and named objects using the same words. Both lexical frequency and phonotactic frequency elicited early, parallel ERP modulations, and importantly this activation pattern was the same for the language production and language perception tasks. Given that across the language modalities, the sensorial input was distinct (visual for the production and auditory for the perception), but the linguistic manipulations were identical, and the rapid parallel effects observed across the modalities cannot be attributed to low‐level input differences. Instead, these findings strongly support the parallel activation of the linguistic components of words.

**Fig. 1:**
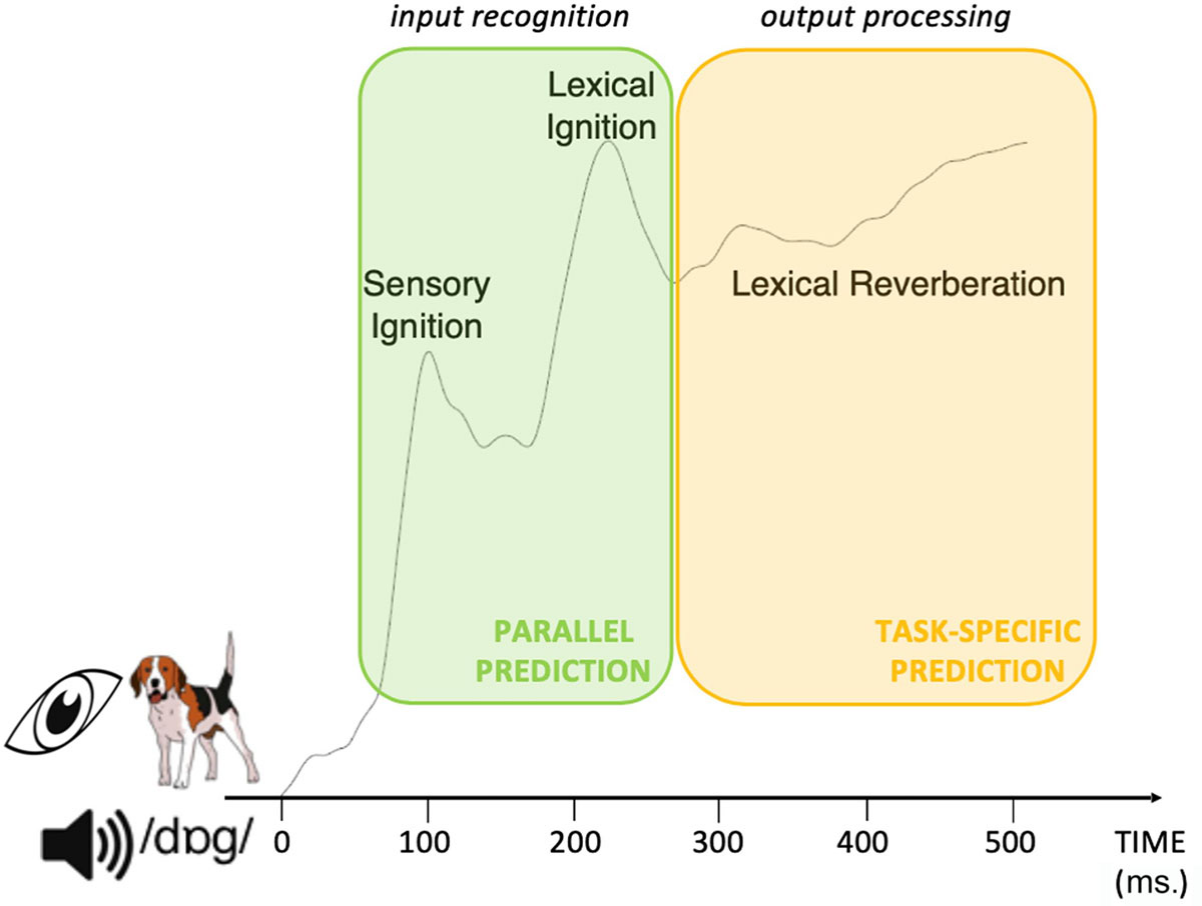
Simplified overview of parallel activation dynamic, where a word (in response to sensorial input: auditory in passive listening tasks and visual in object naming tasks) gets activated rapidly in parallel (ignition phase), and afterward more controlled (and sequential) processing on that ignited word depends on intentions, context and task‐demands (reverberation phase). With such an architecture, prediction‐by‐production will likewise unfold first in parallel, first involving all linguistic components of a word (green), and then by more focused predictions on one or more linguistic components (yellow). (Figure adapted from Fairs et al., 2021.)

It might appear that a parallel activation model has problems with classical findings suggesting a separation between word components, such as the occurrence of semantic or phonological (as opposed to mixed) speech errors (e.g., Dell, 1986; Dell & Raichle, 1981), chronometric evidence from picture‐word interference (e.g., Schriefers et al., 1990; see Levelt et al., 1999), and neurophysiological findings of distinct activation time‐courses for lexical and phonological processing (e.g., Carota, Schoffelen, Oostenveld, & Indefrey, 2022; Indefrey, 2011; Indefrey & Levelt, 2004; Laganaro, Morand, & Schnider, 2009; Sahin et al., 2009; Van Turennout, Hagoort, & Brown, 1997, 1998). However, Strijkers and Costa (2016; see also Pulvermuller, 2002; Pulvermuller et al., 2009) argued that such findings can be explained as task‐ and context‐specific effects emerging after the initial activation of a word representation. Based on the notion that the firing pattern of cell assemblies seems to have two functionally distinct phases, early ignition and later reverberation (e.g., Fuster, 2002; Hebb, 1949; Pulvermuller, 2002), Strijkers and Costa (2016; see also Fairs et al., 2021; Strijkers, 2016) proposed that speech planning follows a similar dynamic: early parallel word retrieval (i.e., recognition or ignition phase), followed by a more controlled and possibly sequential processing phase due to specific context and behavioral requirements (i.e., task‐specific or reverberatory phase; see Fig. 1). If so, lexical access is a rapid parallel process because a word is represented as a stable, integrated assembly (due to the consistent correlations between that word's meaning, grammar, and sounds). However, subsequent processing is more controlled and can focus on specific parts of the word assembly (e.g., meaning) in function of the behavioral goals. This process can explain findings relating to word production that are standardly interpreted in sequential terms. For example, retrieval of a word's semantic and phonological representations would occur in parallel (e.g., Fairs et al., 2021; Strijkers et al., 2017), but meta‐linguistic task‐specific decisions such as identification of specific phonemes of a word (e.g., Van Turennout et al., 1997, 1998) would emerge later.

This dynamic of early parallel recognition and later sequential processing operations is not necessarily restricted to experimental tasks and may form an integral part of language behavior. In everyday communication, words we wish to convey need to be fitted in the appropriate context. To do this, a parallel‐activated word representation must be combined with the representations of other words. Such compositional linguistic operations are context‐dependent and have therefore less stable correlations than is the case for the word representations. As a consequence, many combinatorial operations lack consistent temporal correlations to form integrated assemblies (with the exception of relatively fixed expressions such as idioms; see also Huettig, Audring, & Jackendoff, 2022), and thus rely on more sequential processing to bind different word assemblies together. Take morphological inflection, for example: In the parallel activation model, activating a word occurs in parallel over the levels of representation, but inflectional operations on the activated word, such as making a noun plural or putting a verb in the past tense, are context‐dependent and therefore occur in a more controlled and sequential fashion. In this way, the model accounts for the early effects of word frequency and later effects for morphological inflection (e.g., Sahin et al., 2009; see Strijkers, 2016). This account can similarly explain speech error patterns (e.g., Dell & Raichle, 1981; see Strijkers & Costa, 2016): Most mixed errors (e.g., saying “rat” instead of “cat”) would stem from problems during the parallel activation of the target word (and therefore affect both semantics and phonology), but most semantic‐ or phonology‐specific speech errors (e.g., saying “dog” or “hat” instead “cat”) would be due to later operations on the target word such as the correct sequencing of the phonemes for articulation.

The proposed division between word activation and operation is reminiscent of Hagoort's (2013) MUC (memory, unification, control) model, which distinguishes accessing of memory representations and combinatorial operations on those memory representations (unification). Of course, while Hagoort's model focuses on space (temporal cortex memory, frontal cortex unification), the parallel activation model focuses on time‐course (early memory access and later combinatorial operations). In this manner, the model integrates parallel dynamics with sequential ones (and thus neither negates the existence and need of sequential processing) but attributes functionally different interpretations to these dynamics, compared to traditional models: Lexical access is a parallel process, and task‐ and behavior‐specific processes are sequential. And even though at present most research focused on whether different word components can be accessed in parallel or sequentially, Fairs et al. (2021) did report EEG evidence in line with the notion of rapid parallel recognition followed by sequential task‐specific processing (see Fig. 1). The authors observed the same early parallel effects for lexical and phonotactic frequency in production and perception, but different processing dynamics between the language modalities later, and interpreted these differences as due to the distinct task requirements for production (overt object naming) and perception (semantic categorization). Such results provide an interesting starting point to study the potential interplay of parallel and sequential processing. For the remainder of this article, we consider the implications of a parallel activation model of language production for language prediction, and then specifically the notion of prediction‐by‐production.

### 1.2 Using language production to predict words in a parallel activation model

There is much evidence that comprehenders predict what they are likely to hear or read and that such prediction takes place at different levels of representation (Huettig, 2015; Kuperberg & Jaeger, 2016). For example, Altmann and Kamide (1999) found that comprehenders tended to look at a picture of a cake after hearing *The boy will eat*, suggesting that they predicted that an edible object would soon be mentioned. Many other studies support prediction of meaning (e.g., Federmeier & Kutas, 1999; Grisoni, Miller, & Pulvermuller, 2017), grammar (e.g., Strijkers et al., 2019; Van Berkum, Brown, Zwitserlood, Kooijman, & Hagoort, 2005; Wicha, Moreno, & Kutas, 2004), or sound (e.g., DeLong et al., 2005; Ito, Pickering, & Corley, 2018; but see Nieuwland et al., 2018).

Recent research suggests that comprehenders use their production systems to predict what their interlocutor may say next (see Pickering & Gambi, 2018). For example, Martin, Branzi, and Bar (2018) had Spanish‐speaking participants read highly predictive sentence contexts followed by either expected or unexpected words that differed in grammatical gender (e.g., *El rey llevaba en la cabeza una corona/un sombrero*; “The king wore on his head a crown/a hat”). An unexpected word leads to an N400 effect relative to an expected word (e.g., Kutas & Hillyard, 1984), and an article or adjective whose gender is not consistent with the expected word also leads to an N400 effect, suggesting that comprehenders predict gender (Van Berkum et al., 2005; Wicha et al., 2004). But Martin et al. found that the N400 response to articles whose gender was unexpected was eliminated when participants simultaneously performed an articulatory suppression task that taxed the production system (i.e., repeatedly pronouncing a syllable) as compared to two control conditions (i.e., tongue tapping, listening to a recording of one's voice pronouncing the syllable). Thus, inhibiting the production system appeared to interfere with prediction.

In addition, Lelonkiewicz, Rabagliati, and Pickering (2021) had participants name a picture whose name corresponded to the expected word following a highly predictive context or to a possible word following an unpredictive context. This led to an effect of predictability on picture naming. In one condition, participants read the context aloud—a manipulation that was designed to activate the production system. In this condition, the predictability effect was enhanced, compared to silent reading. Thus, activating the production system appeared to enhance prediction. Together, these studies suggest that comprehenders use their production system to generate predictions.

Pickering and Gambi (2018) proposed an account of prediction‐by‐production, in which people first comprehend a predictable context (e.g., *The boy went outside to fly a …*) and then convert the representations into ones they would use if they were producing it. They use these representations to derive the intention that they would use to continue the speaker's utterance. They then apply “self‐other adjustments” to compensate for differences between themselves and the speaker in relation to memory for the linguistic context and access to the non‐linguistic context—essentially, so they predict what they believe the speaker would say rather than what they themselves would say. For example, a male comprehender who heard a female speaker utter *I would like to wear the nice …* would be likely to predict a stereotypically feminine word such as *dress* even if he would himself be likely to utter a stereotypically masculine word such as *tie* (Corps, Brooke, & Pickering, 2022). They now predict the upcoming word by going through the stages involved in language production but without actually articulating.

**Fig. 2:**
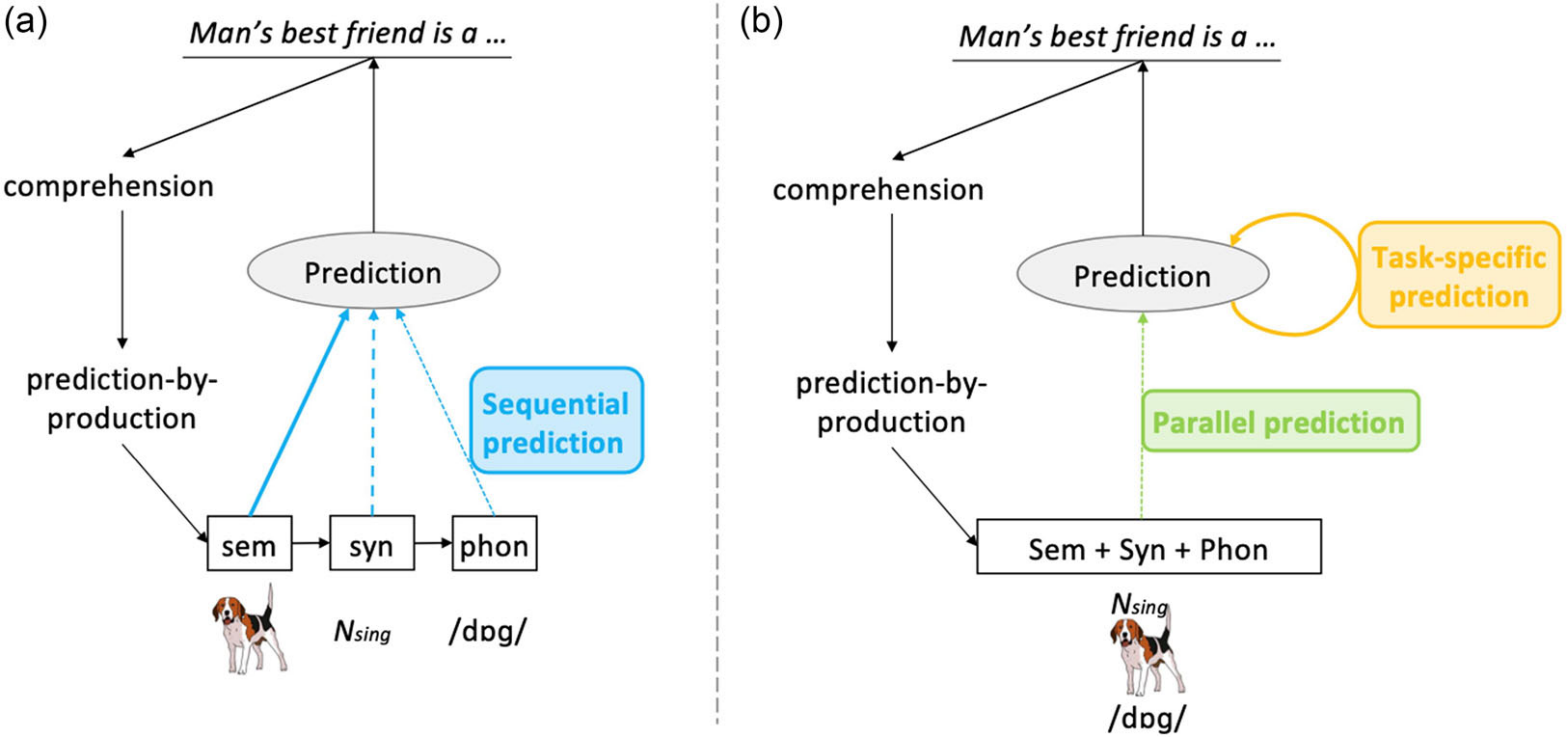
Schematic contrast of prediction‐by‐production in a sequential (a) versus parallel (b) model. When hearing a sentence like “*Man's best friend is a…*” in a prediction‐by‐production mechanism, comprehenders covertly imitate their interlocutor and then use their production system to estimate the most likely continuation (here: “*dog*”). Within a sequential lexical production architecture (a‐panel), this means they predict the semantics (sem) before the syntax (syn) before the phonology (phon), and similarly the strength of prediction follows that same sequence (strongest for semantics, then syntax, and last phonology; depicted here by the thickness of the blue arrows; for more details, see Pickering & Gambi, 2018). In contrast, within a parallel production model (b‐panel), comprehenders first simultaneously activate all linguistic components of the predicted word continuation in (green arrow) and subsequently enhance the activation of those predicted components that are most relevant for their task and behavioral goals (yellow arrow).

Pickering and Gambi (2018) assumed a traditional serial account of language production (e.g., Levelt et al., 1999). Semantic prediction should occur first and be most likely to occur. Grammatical prediction should occur next and be next most likely to occur. Phonological prediction should take the longest to occur and would occur least often (see Fig. 2a) In support of these claims, Pickering and Gambi pointed out that many studies find strong and reliable prediction of meaning (e.g., Altmann & Kamide, 1999), whereas prediction of form occurs in some studies (e.g., DeLong et al., 2005; Ito et al., 2018) but not others (e.g., Amos, Seeber, & Pickering, 2022; Nieuwland et al., 2018). Thus, comprehenders might not predict later representations such as phonology before the speaker utters the next word. Moreover, language production is effortful, so making predictions based on production is also effortful. For example, comprehenders might assign resources to producing a quick response during conversation (given that gaps between turns are often around 200 ms; e.g., Levinson, 2016), and therefore not be able to make phonological predictions.

But if we replace this account with the parallel account we described in the previous section, prediction‐by‐production makes different claims (see Figs. 1 and 2b). One such notable difference is prediction flexibility. Since in a parallel system the meaning, grammar, and sounds of a word are available at the same time (i.e., ignition phase), a speaker can choose which linguistic component (or all) to focus prediction on as a function of the goal‐directed behaviour (i.e., reverberation phase). Consequently, the prediction of one component (e.g., sounds) is not dependent on the prediction of another component (e.g., meaning) as is the case in a sequential prediction‐by‐production mechanism. A parallel prediction‐by‐production mechanism shares this tenet of flexibility with the parallel architecture (PA) model of linguistic processing (e.g., Jackendoff, 2007), and its recent implementation to explain predictive language processing (Huettig et al., 2022). Because in PA the three strata (meaning, grammar, and sound) are independent processors, prediction in one stratum is not dependent on prediction in the other strata. And even though the PA model and a parallel activation version of prediction‐by‐production have different underlying structures to support such flexibility (in PA because of the independence of representations and in parallel activation because of the integration over representations making them simultaneously available), neither is bound to the strict hierarchy of processing steps found in a traditional sequential model.

**Fig. 3:**
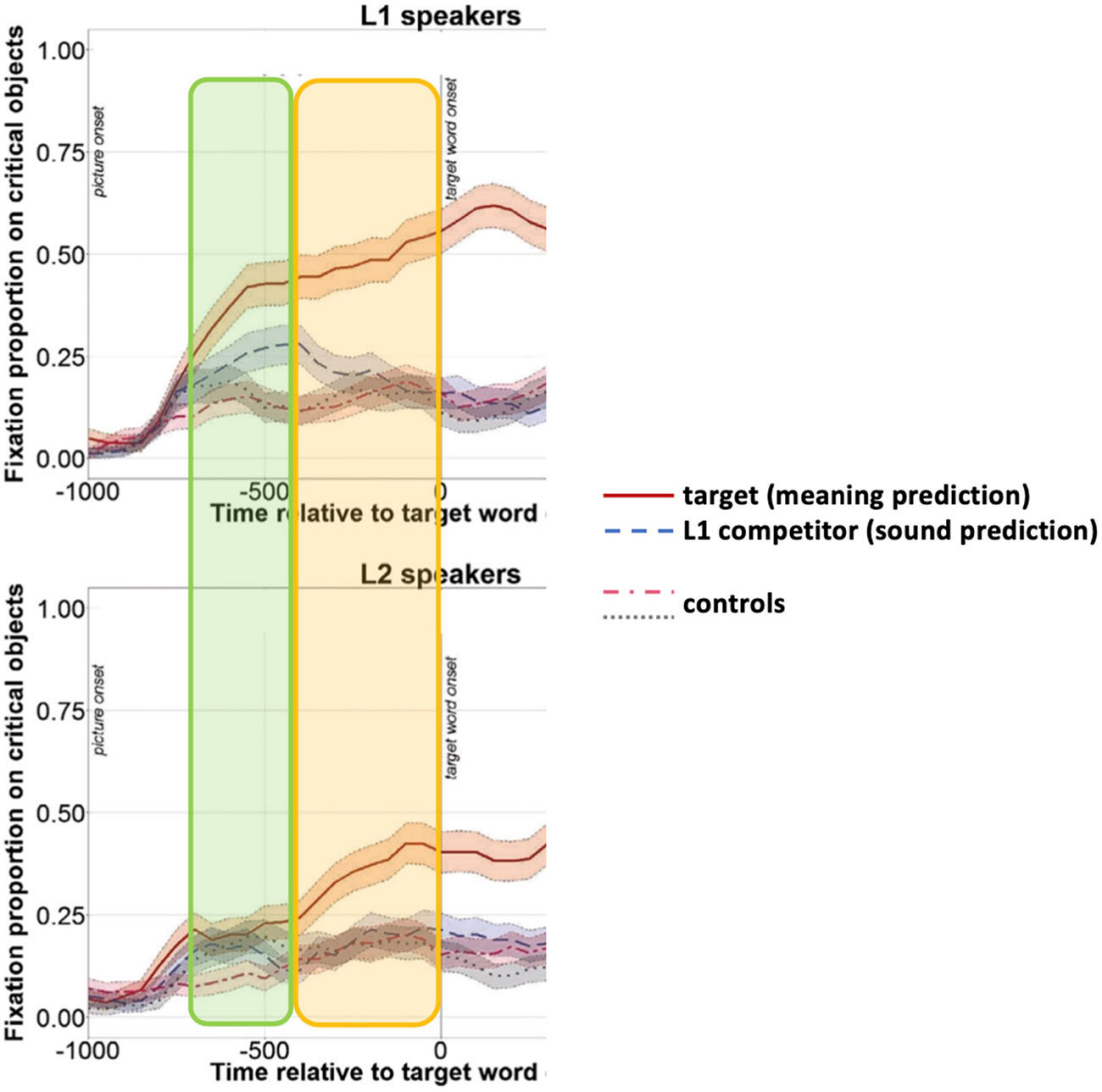
Example to explain predictive eye‐movement data from a visual world paradigm (Ito et al., 2018) within a parallel prediction‐by‐production architecture. When hearing a sentence (e.g., “*Man's best friend is a…*”), we see that both L1 and L2 speakers make predictive eye movements to a target object (e.g., “*dog*”), and this occurs earlier in L1 speakers (red line significantly different from control lines before target word onset). For a display where the target is not present, but a phonological competitor is (e.g., “*doll*”), L2 speakers show no sign of predictive eye movements, but L1 speakers do, though this effect is much smaller in magnitude and duration (blue line being different from controls). Within a parallel prediction‐by‐production architecture, the full pattern of results is explained by assuming L1 speakers (who have strong memory representations) make stronger parallel predictions (green area showing both meaning and sound predictions), while L2 speakers (weaker memory representations) need the more controlled task‐specific predictions to predict word meaning (yellow area). (Figure adapted from Ito et al., 2018.)

Let us demonstrate this idea based on the predictive eye‐movement data reported by Ito et al. (2018). Using a visual world paradigm, the authors observed that both native and non‐native English speakers made predictive eye movements to an object denoting the likely final word of a sentence (indicating semantic prediction), but only native speakers also made predictive eye movements to a phonological competitor (indicating phonological prediction; see Fig. 3). The result was taken as evidence that semantic predictions were faster and stronger than phonological predictions because the non‐native speakers did not have sufficient resources to make phonological predictions. For example, after hearing “man's best friend is a,” participants predict “dog,” and subsequently its phonological form /dɒɡ/, which then activates phonologically related word forms such as /dɒl/. (Note that this account assumes they predict the phonology associated with “dog,” even though they may have ascertained that there is no dog in the display.)

In contrast, in a parallel prediction‐by‐production system, phonological predictions affecting processing in the absence of semantically related information are expected since meaning and sounds are available at the same time (i.e., word ignition phase) (see Fig. 3). These phonological predictions are short‐lived and less robust than semantic predictions because the task primarily involves judging semantics, and prediction will quickly focus on meaning rather than sounds (i.e., task‐specific reverberation; see Fig. 3). Consequently, a parallel prediction‐by‐production system would suggest that if we alter the task to emphasize sounds (e.g., rhyme judgment), phonological predictions should become stronger, compared to the semantic task. This shift in focus may also make phonological predictions viable for weaker memory representations, such as those in non‐native speakers. In contrast, a sequential prediction mechanism cannot accommodate such flexibility.^2^ The idea of prediction flexibility provided by a parallel linguistic architecture offers an interesting and promising alternative to the sequential implementation and warrants further investigation. In sum, we have argued that lexical access during speech planning is compatible with a PA and applied this radical account to prediction.

## Notes

1 By consistent co‐occurrence, we refer to the combination of the meaning‐, grammar‐, and sound‐features of a word, for example, the combination of “round, bouncing, toy” + “noun” + “/bɔl/” when there is the intention to utter the word “*ball*.” It does not mean that those features in isolation uniquely co‐occur. A homophone like *“ball”* can link to different meanings (e.g., dancing event) and would thus constitute a different word assembly (and the combination of the meaning “dancing event” + the sounds “/bɔl/” would also be a consistent co‐occurrence). In this way, a parallel activation model can explain processing differences between homophonous words and non‐homophonous words (and similarly for synonyms, hyperonyms, etc.; for more detail see, e.g., Strijkers, 2016).

2 Note that there are also other, non‐mutually exclusive prediction mechanisms, such as forward models (e.g., Pickering & Garrod, 2013; Runnqvist et al., 2021) or prediction‐by‐association (e.g., Pickering & Gambi, 2018), which can account for some of the phenomena we discuss, but the objective here is to focus on prediction‐by‐production and contrast such mechanism in a sequential versus PA.
